# Research on the Deformation Law of Foundation Excavation and Support Based on Fluid–Solid Coupling Theory

**DOI:** 10.3390/s24020426

**Published:** 2024-01-10

**Authors:** Rongyu Xia, Zhizhong Zhao, Risheng Wang, Maolin Xu, Shujun Ye, Meng Xu

**Affiliations:** 1 School of Transportation Civil Engineering, Shandong Jiaotong University, Jinan 250357, China; 2 Shandong Dongtai Engineering Consulting Co., Zibo 255000, China

**Keywords:** excavation support, fluid–structure interaction, wireless sensors, finite element simulation, deformation patterns, early warning thresholds

## Abstract

To investigate the impact of underground water seepage and soil stress fields on the deformation of excavation and support structures, this study initially identified the key influencing factors on excavation deformation. Subsequently, through a finite element simulation analysis using Plaxis, this study explored the effects of critical factors, such as the excavation support form, groundwater lowering depth, permeability coefficient, excavation layer, and sequence on excavation deformation. Furthermore, a comprehensive consideration of various adverse factors was integrated to establish excavation support early warning thresholds, and optimal dewatering strategies. Finally, this study validated the simulation analysis through an on-site in situ testing with wireless sensors in the context of a physical construction site. The research results indicate that the internal support system within the excavation piles exhibited better stability compared to the external anchor support system, resulting in a 34.5% reduction in the overall deformation. Within the internal support system, the factors influencing the excavation deformation were ranked in the following order: water level (35.5%) > permeability coefficient (17.62%) > excavation layer (11.4%). High water levels, high permeability coefficients, and multi-layered soils were identified as the most unfavorable factors for excavation deformation. The maximum deformation under the coupled effect of these factors was established as the excavation support early warning threshold, and the optimal dewatering strategy involved lowering the water level at the excavation to 0.5 m below the excavation face. The on-site in situ monitoring data obtained through wireless sensors exhibited low discrepancies compared to the finite element simulation data, indicating the high precision of the finite element model for considering the fluid–structure interaction.

## 1. Introduction

As the construction of transportation infrastructure steadily progresses, the stability of the excavations, which serve as the fundamental guarantee of engineering quality, has gradually become a focal point in the field of construction [1,2]. Faced with diverse architectural structures, it is essential to select excavation forms of varying scales to meet construction requirements. However, due to the differing geological conditions in various regions and the lag in corresponding theoretical system updates, frequent accidents during excavations have occurred [3,4,5]. Through a comprehensive analysis of various excavation accidents, two main types were identified: one is the collapse of the excavation itself, caused by the instability of the excavation support structure [6,7,8], and the other is the overturning of the excavation due to excessive settlement around the excavation [9,10,11]. A further analysis of the destruction mechanisms based on the accident type revealed that approximately 90% of excavation safety issues are attributed to the mutual influence of excavation seepage and soil consolidation [12,13,14]. The construction of deep excavations is typically accompanied by dewatering, altering the groundwater seepage field and the stress field of the surrounding soil. This leads to changes in the displacement field of the soil inside and outside of the excavation, ultimately causing the deformation of the excavation support structure. When the groundwater level is too high, water infiltrates the excavation through the seepage field, causing soil instability and failure. Conversely, when the groundwater level is too low, the soil inside the excavation consolidates and hardens, altering the stress field and resulting in settlement around the excavation [15]. Numerous scholars worldwide have conducted numerical simulation studies on excavation construction. Lin [16] quantitatively simulated the stress–strain state of the soil, delving into the deformation patterns of excavation under different excavation methods. Koutsofta [17] studied the impact of two-dimensional seepage using the case of the Boston Tower deep excavation project. Zhang Shuibing [18] simulated the deformation of support structures during reverse construction using finite element software, revealing a minimal impact on the displacement of the support structures. Cai Jiangning [19] utilized a finite element simulation to study excavation, explaining the regularities between soil settlement and excavation depth around the excavation. While stress–strain simulation methods for soil and rock are relatively mature, research on coupled water–soil interactions and seepage are still in their early stages, making it challenging to accurately simulate the dynamic changes in water levels that occur during dewatering excavation, and the results may lack precision. Therefore, it is necessary to comprehensively consider the impact of the fluid–structure coupling mechanism on the deformation of excavation supports.

Previous research has indicated that the primary factors influencing excavation deformation include the excavation geometry, support forms, seepage inflow rates, excavation soil, and excavation sequences [20,21,22,23,24,25,26,27]. Some scholars have optimized excavation design schemes through quantitative studies on individual influencing factors [28,29,30,31,32], providing new insights into excavation construction processes. However, these studies have often overlooked the mutual coupling effects of other excavation factors. Other scholars have summarized the patterns of excavation deformation through numerical simulations [33,34,35,36], offering rich theoretical guidance for excavation deformation, but lacking validation through actual engineering data. Therefore, this study comprehensively summarizes the deformation patterns of excavations under fluid–structure coupling and provides an all-encompassing theoretical and technical support for excavation construction safety. Firstly, based on fluid–structure coupling theory, simulation models of the excavation support forms, groundwater lowering depths, permeability coefficients, excavation soil layers, and excavation sequences were developed using Plaxis finite element software. Secondly, a combination of various adverse factors were integrated to explore excavation support early warning thresholds and optimal dewatering strategies. Finally, relying on on-site in situ testing at the Anhui Damao Interchange Project, a comparison analysis was conducted for the data collected through the wireless sensors and the finite element simulation data, to validate the accuracy of the numerical simulations and summarize the patterns of changes.

## 2. Project Profile

**Figure 1:**
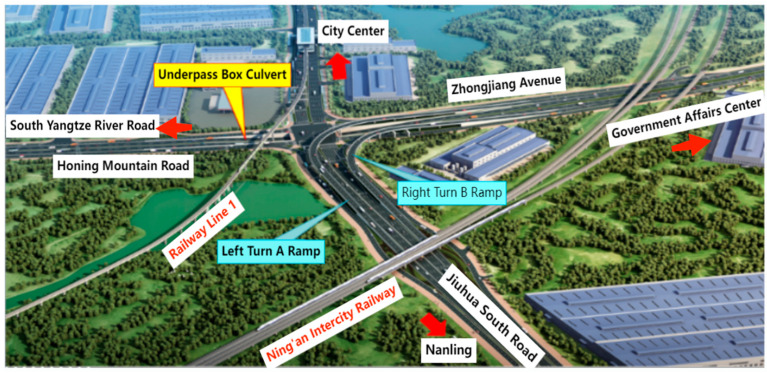
Project location.

**Figure 2:**
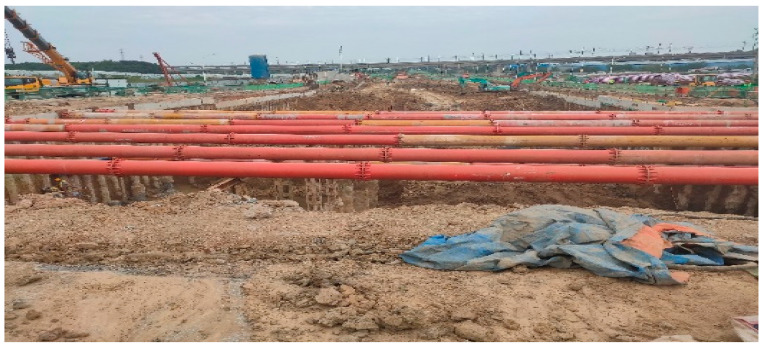
Real location.

The Damao Interchange Project is located in Wuhu City, Anhui Province, covering the construction area of Zhongjiang Avenue, Jiuhua South Road, and the roadside auxiliary lanes. Zhongjiang Avenue passes underneath Jiuhua South Road, employing a U-shaped trench combined with an integral box culvert structure. The entire structure is divided into 15 segments, with segment lengths of 6 × 30 + 2 × 34 + 15 + 18 + 4 × 30 + 15 m. The total length of the integral box culvert is 68 m, and the deepest part of the culvert foundation is 14.5 m. Figure 1 shows the project location, and Figure 2 depicts a real-life scene.

According to geological survey reports and local hydrological data, the hydrological conditions around the site are complex, with a network of intersecting channels and ponds. The surface water is mainly in ditches and ponds, and it is significantly influenced by seasonal variations in precipitation. This could have adverse effects on the project’s construction. Therefore, protective measures must be taken during the excavation and support processes. Timely actions, such as excavation clearing or pile reinforcement, should be implemented based on geological reports and local hydrological data.

## 3. Modeling

### 3.1. Selection of Parameters

To elucidate the influence patterns of the various factors on the deformation of excavation support systems under fluid–structure coupling, this study, in conjunction with the Damao Interchange Project in Anhui Province, established a finite element model for excavation support by Plaxis 2D Connect Edition V20 software, which is developed by PLAXIS B.V. Company in Delft, Netherlands. The simulation analysis focused on the deformation of the excavation under the influence of key control parameters during the excavation support process, including the excavation support form, groundwater lowering depth, permeability coefficient, excavation layer, and excavation sequence. The values for each parameter were determined through a combination of on-site exploration and laboratory experiments.

(1) Excavation Support Form

Two commonly used excavation support systems were selected for the numerical simulation: the external anchor support system and the internal pile support system.

(2) Groundwater Lowering Depth

Groundwater levels of 2 m, −5 m, and −8 m were chosen, corresponding to the scenarios of heavy rain, moderate rain, and light rain.

(3) Permeability Coefficient

The soil’s permeability coefficients were set at 0.005 m/day, 0.04 m/day, and 0.08 m/day, representing the unsaturated, semi-saturated, and fully saturated states of the soil.

(4) Excavation Size

Excavation dimensions were set as follows: 5 m × 5 m, 10 m × 10 m, 10 m × 20 m, and 15 m × 30 m (depth × width).

### 3.2. Defining Materials

Based on the actual conditions of the Damao Interchange Project in Wuhu City, Anhui Province, various material parameters were defined using indoor experiments, including water content tests, density tests, liquid limit tests, compaction tests, and triaxial tests, in conjunction with geological survey reports. The specific parameters are detailed in Table 1, Table 2 and Table 3.

### 3.3. Boundary Conditions

Based on fluid–structure coupling theory, two types of boundaries were defined: one for seepage and the other for mechanics. For the seepage boundary, only the influence of rainfall infiltration on the deformation of the excavation was considered. Therefore, the upper part of the model was set as a permeable layer, while the sides and bottom were set as impermeable layers. For the mechanical boundary, fixed constraint boundary conditions were applied to the bottom of the excavation, no boundary conditions were applied to the top, and normal stress constraint boundary conditions were applied to the other four sides [37,38].

## 4. Simulation Analysis

### 4.1. Analysis of Factors Influencing the Deformation Patterns of Excavations

#### Influence of Excavation Support Forms on the Deformation Patterns of Excavations

**Figure 3:**
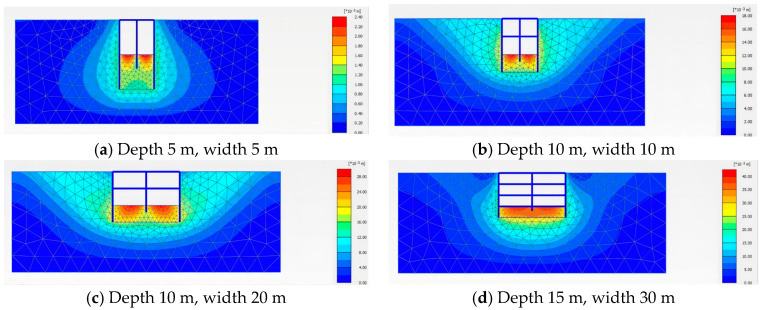
Cloud diagram of the support model within the pile of different sizes.

**Figure 4:**
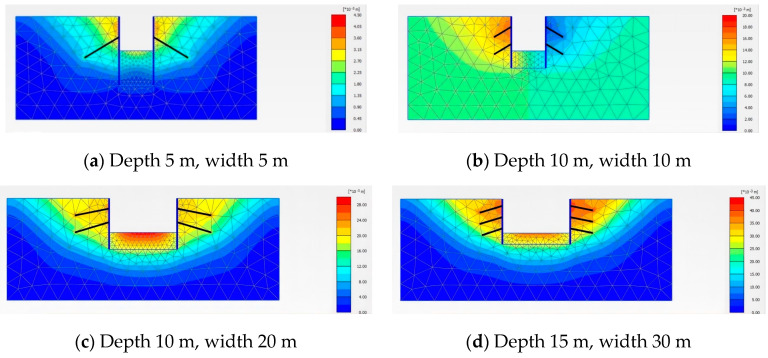
Cloud diagram of outer support model for different sizes of anchor rods.

**Figure 5:**
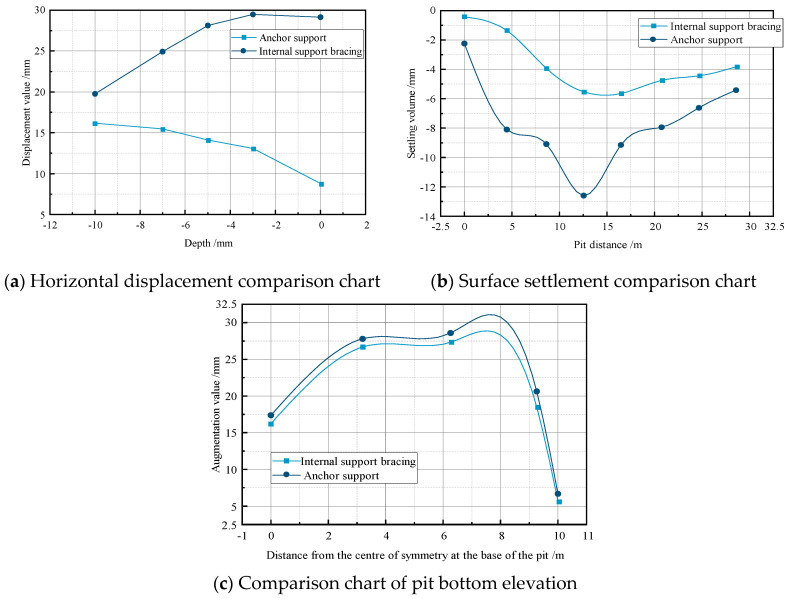
Comparison of support system structure.

We conducted a deformation analysis of two different excavation support systems, maintaining a constant excavation water level of −5 m and a permeability coefficient of 0.04 m/day. Figure 3 and Figure 4 depict the deformation contour maps of the excavations supported by the internal pile support system and the external anchor support system, respectively. Figure 5, using a 10 m × 20 m excavation model as the example, compares the horizontal displacement, surface settlement, and bottom heave deformation of the structures under the two excavation support forms.

From the figures, it is evident that the excavation supported by the internal pile support system exhibits better deformation stability compared to the external anchor support system. At each measurement point, the corresponding maximum horizontal displacement, surface settlement, and bottom heave for the internal pile support system are 16.176 mm, −5.15 mm, and 27.516 mm, respectively. The maximum horizontal displacement for the external anchor support system is 28.924 mm, indicating an increase of 12.748 mm compared to the internal pile support system, with a growth rate of 44%. The maximum settlement for the internal pile support system is −5.15 mm, while for the external anchor support system it is −11.797 mm, representing an increase of 6.647 mm, or 56.3%. The maximum heave for the internal pile support system is 27.516 mm, and for the external anchor support system it is 28.631 mm, showing an increase of 1.115 mm, or 4%, compared to the internal pile support system.

From the simulations of these two different excavation support systems, it can be concluded that in terms of the overall deformation, the different support forms have a significant impact on the horizontal displacement of the support structure and surface settlement, while the influence on the bottom heave is relatively small. The internal pile support system exhibits better stability, with an overall reduction in the excavation deformation of 34.5% compared to the external anchor support system. Therefore, for this project, the internal pile support form should be prioritized, and all the following studies are based on the internal pile support system.

### 4.2. Influence of Water Level on the Deformation of Excavations

**Figure 6:**
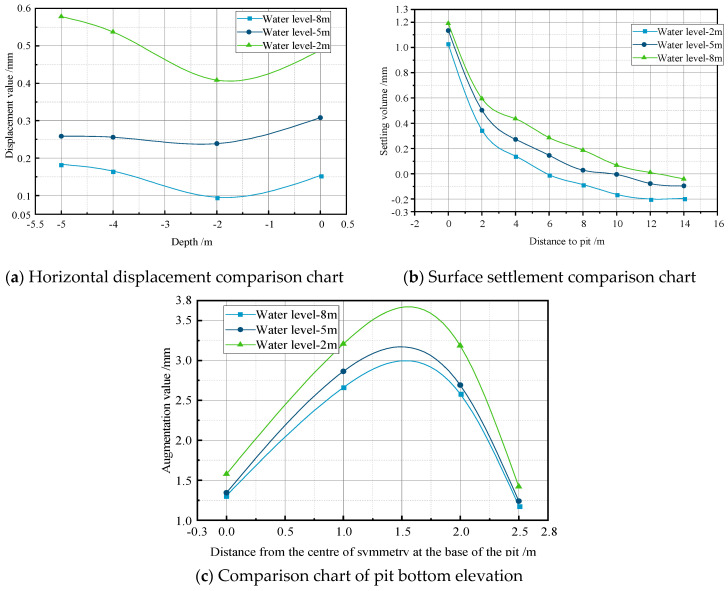
Comparative analysis of water level on support system within the pile.

To investigate the impact of the water level under coupled field conditions on the deformation of excavations, a simulated analysis was conducted on an excavation supported by an internal pile support system, with dimensions of 5 m × 5 m. Throughout the process, the permeability coefficient was kept constant at 0.04 m/day, while the water levels were adjusted to −2 m, −5 m, and −8 m. Based on the simulation results, a comparative analysis of the horizontal displacement, surface settlement, and bottom heave of the excavation support structure at different water levels was performed, as shown in Figure 6.

When the water level was −2 m, the maximum values for the horizontal displacement, surface settlement, and bottom heave at the measurement points were observed, corresponding to 0.57 mm, −0.198 mm, and 3.212 mm, respectively. As the water level gradually decreased from −2 m to −8 m, the maximum horizontal displacement decreased by 0.397 mm, representing a reduction of 69.6%. The maximum settlement decreased by 0.165 mm, showing a reduction of 83.3%. The maximum heave decreased by 0.556 mm, indicating a reduction of 17.3%. This suggests that when the permeability coefficient remains constant, the horizontal displacement, surface settlement, and bottom heave are positively correlated with the water level, and the water level has the greatest impact on the surface settlement. In practical engineering, it is advisable to choose a lower water level.

### 4.3. Influence of the Permeability Coefficient on Excavation Deformation

**Figure 7:**
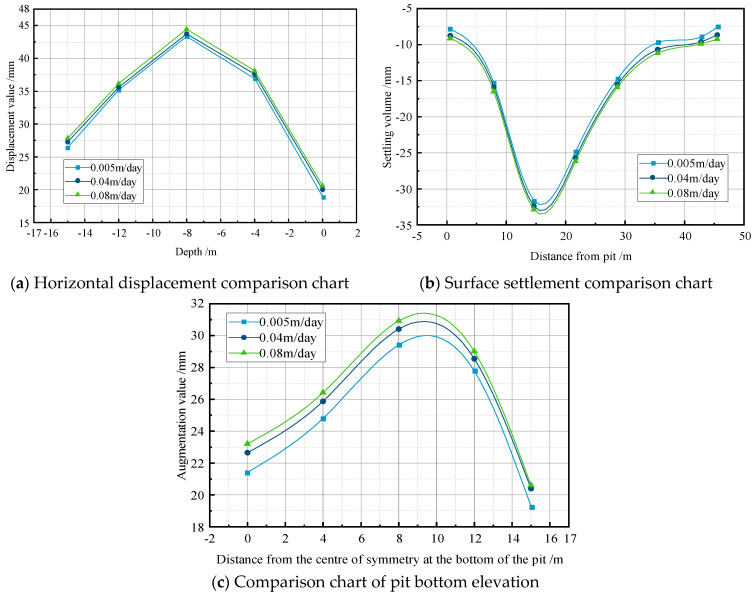
Comparative analysis of permeability coefficient on support system within the pile.

To investigate the impact of the permeability coefficient on the deformation, simulations were conducted on a pile-supported excavation with dimensions of 15 m × 30 m, while maintaining a constant water level of −2 m. The permeability coefficient was varied at rates of 0.005 m/day, 0.04 m/day, and 0.08 m/day. The simulation results were then analyzed to compare the excavation support structure’s horizontal displacement, surface settlement, and bottom heave under the different permeability coefficients, as illustrated in Figure 7.

When the permeability coefficient was 0.08 m/day, the maximum values for the horizontal displacement, surface settlement, and bottom heave were 44 mm, −32.904 mm, and 31.014 mm, respectively. As the permeability coefficient gradually decreased from 0.08 m/day to 0.005 m/day, the maximum values for the horizontal displacement, surface settlement, and bottom heave exhibited a decreasing trend, with reduction percentages of 2.6%, 3.2%, and 4.9%, respectively. The analysis indicates that, while maintaining a constant water level, an increase in the permeability coefficient leads to a larger horizontal displacement, surface settlement, and bottom heave of the excavation support structure. The permeability coefficient, in particular, influences the force causing bottom heave. In actual construction processes, the real-time exploration of the excavation soil is necessary for adjusting the permeability coefficient calculations.

### 4.4. Influence of Soil Excavation and Sequence on Excavation Deformation

**Figure 8:**
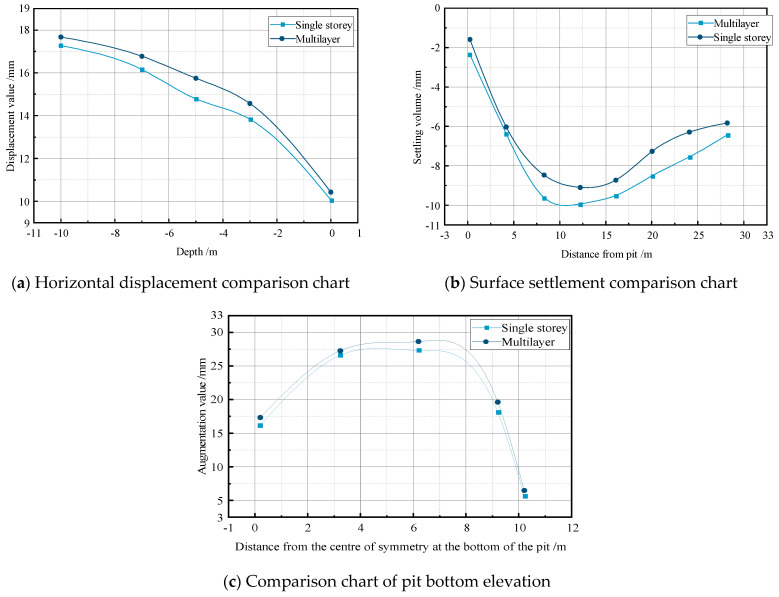
Comparative analysis of excavation of soil layer of support system within the pile.

In deep excavation support systems, different soil layers are often encountered. In consideration of this, simulations were conducted on a pile-supported excavation with dimensions of 10 m × 20 m. The water level was maintained at −2 m, and the permeability coefficient was kept constant at 0.04 m/day. The simulations included scenarios with the excavation of a single soil layer and multiple soil layers, with variations in the excavation and support sequence. The simulation results are presented in Figure 8.

As observed in the figure, excavating multiple soil layers results in a significantly larger deformation in the excavation. When excavating a single soil layer, the maximum horizontal displacement, surface settlement, and bottom heave are 17.104 mm, −9.049 mm, and 27.516 mm, respectively. In the case of excavating multiple soil layers, these values increase to 17.546 mm, −9.873 mm, and 28.745 mm, with percentage increases of 2.6%, 9.1%, and 4.5%, respectively. The results indicate that the type of excavated soil layer has a relatively minor overall impact on the excavation deformation. However, the interaction of forces between the soil layers during the excavation process can lead to significant effects on the surface settlement. Therefore, the influence of the excavated soil layers on the excavation deformation should be carefully considered in actual.

Regarding the analysis of the excavation sequence’s impact, the model was initially configured with the excavation and support stages swapped, i.e., excavation followed by support. However, the finite element simulations revealed convergence issues, even under conditions with small dimensions, low water levels, and a low permeability coefficient. This is attributed to the destabilization of the excavation due to groundwater seepage, which causes the surrounding soil to slide into the pit, and results in instability and collapse. Hence, it is imperative to adhere to the principle of support before excavation in actual construction scenarios.

### 4.5. Establishment of Excavation Support Warning Thresholds

#### Identification of the Most Adverse Factors for Deep Foundation Excavation

The simulation results reveal the following trends: as the water level rises from −8 m to −2 m, the overall deformation of the excavation increases by 35.5%. Similarly, an increase in the permeability coefficient from 0.005 m/day to 0.08 m/day leads to a 17.62% overall increase in the excavation deformation. Transitioning from a single-layered soil excavation to a multi-layered soil excavation results in an overall increase in the excavation deformation of 11.4%. The impact of the relationships among the various factors influencing excavation deformation can be ranked as follows: water level > permeability coefficient > excavation soil layer. Among these factors, high water levels, elevated permeability coefficients, and the presence of multiple soil layers are identified as the most adverse conditions for deep foundation excavation support.

### 4.6. Warning Threshold

**Figure 9:**
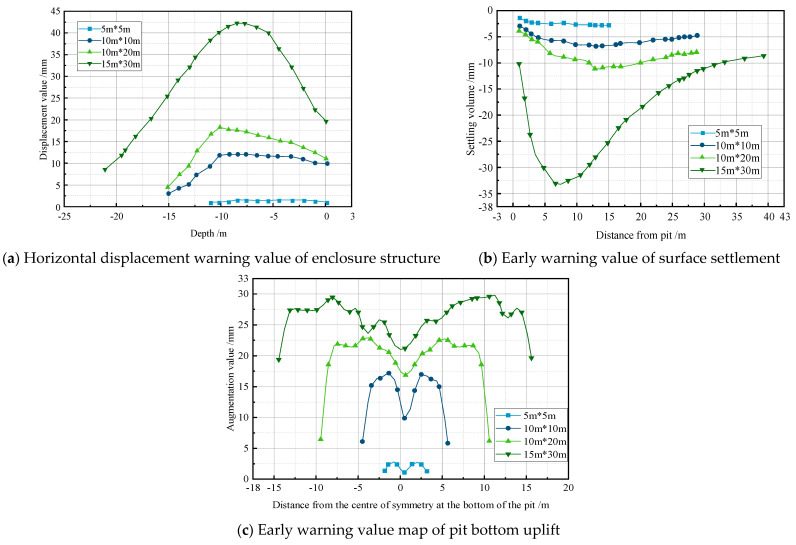
Map of early warning values for different sizes of pile body support systems.

When multiple factors, such as high water levels, permeability coefficients, and multi-layered soil excavation coexist, the maximum value obtained from the finite element simulations serves as the critical threshold for excavation failure. This critical threshold, derived from the simulation results, can be utilized as the warning value under the influence of fluid–structure coupling, providing crucial guidance for subsequent engineering processes. The deformation warning values for excavations of different sizes are illustrated in Figure 9.

### 4.7. Optimizing Dewatering Strategies

The depth of precipitation is the most critical factor influencing safety issues during foundation pit excavation. Typically, the water level inside the pit is lowered to a depth ranging from 0.5 m to 2 m below the excavation surface before excavation. Different precipitation depths have varying effects on the deformations of a foundation pit’s retaining structures. An inadequate precipitation depth fails to keep the excavation surface dry, while an excessively high precipitation depth leads to a significant water head difference inside and outside of the pit. In actual construction practice, the selection of different precipitation schemes and the effective control of the excavation and support system within the warning range are crucial challenges that must be addressed. Therefore, this section simulates different precipitation depths, lowering the groundwater level to 0.5 m, 1 m, 1.5 m, and 2 m below the excavation surface, with an initial groundwater level of −2 m. Keeping the other conditions constant, the impact of the different precipitation depths on the foundation pit deformations is analyzed to provide optimal dewatering strategies for subsequent construction stages.

**Figure 10:**
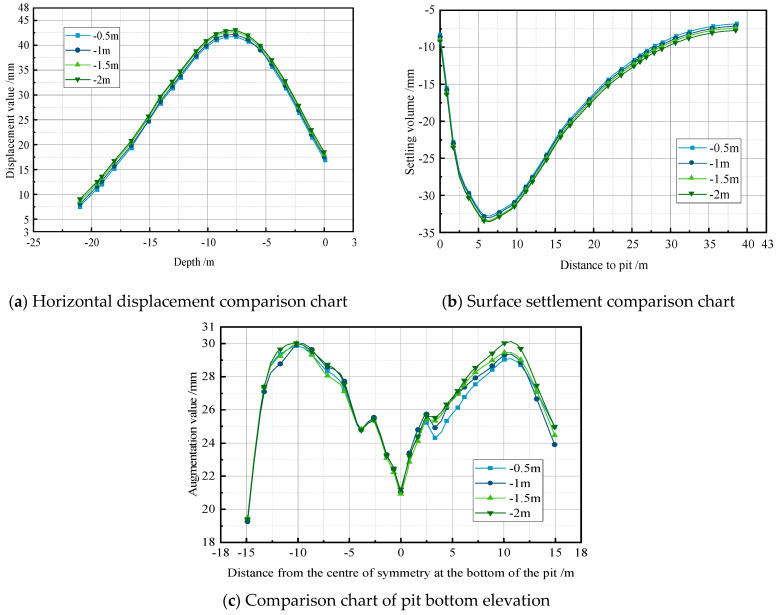
Precipitation depth analysis map.

Through a comparative analysis of the foundation pit variations under different precipitation depth conditions (Figure 10), it is observed that as the pit’s dewatering depth increases from 0.5 m to 2 m, the deformation of the foundation pit increases further. The horizontal displacement of the retaining structure increases by approximately 3.1%, the surface settlement increases by about 2.3%, and the pit bottom heave increases by around 0.5%. With an increase in precipitation depth, there is a proportional rise in the horizontal displacement of the support piles, surface settlement, and pit bottom heave. The analysis suggests that increasing the precipitation depth results in a larger deformation due to the gradual increase in the water head difference inside and outside of the pit. In summary, lowering the water level inside the pit to 0.5 m below the excavation surface is the optimal approach, providing a feasible solution for the subsequent on-site construction phases.

## 5. Verification Using Actual Engineering Projects

**Figure 11:**
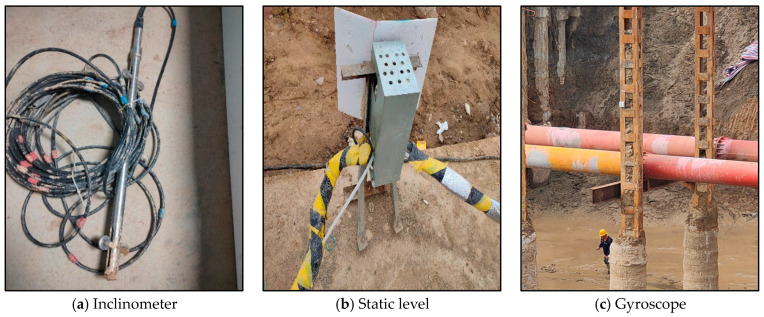
Sensor.

**Figure 12:**
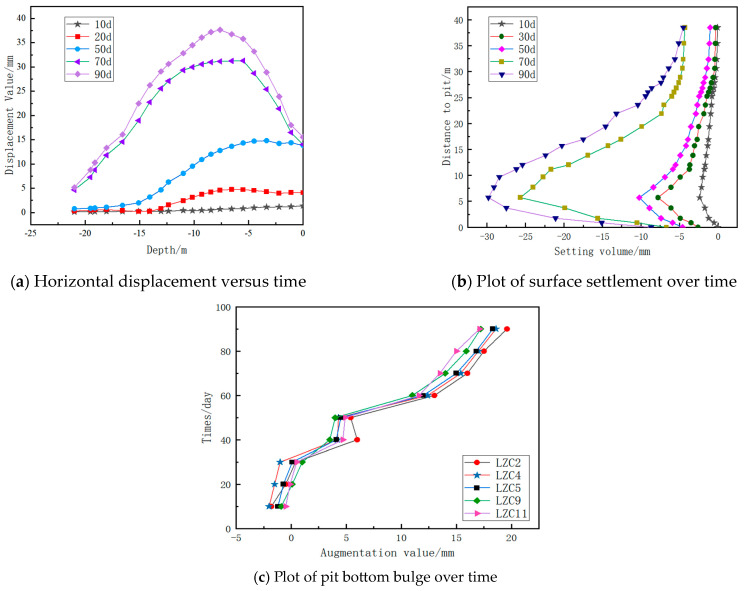
Sensor monitoring data analysis graph.

**Figure 13:**
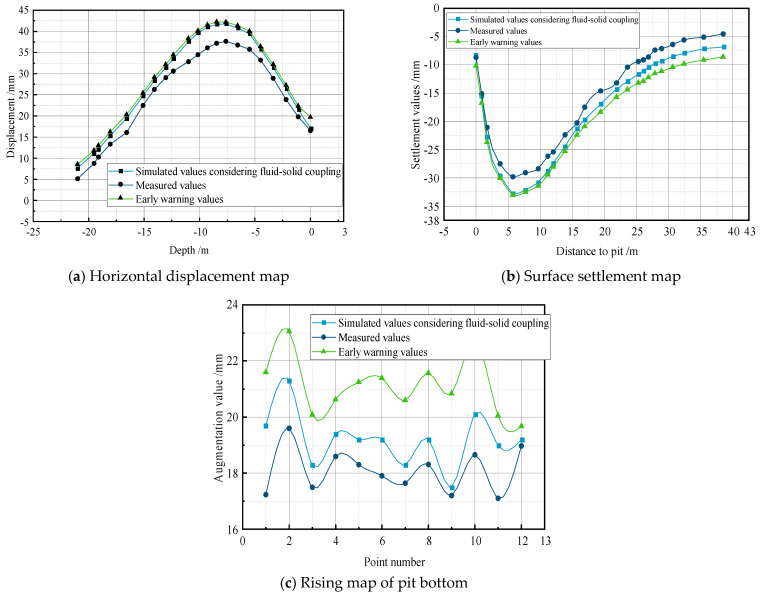
Comparison chart of project’s actual measurements.

Based on the previous finite element simulation study of excavation and support processes in deep foundations, the deformation patterns of the excavation soil and support structures under different conditions were obtained, providing a theoretical basis for monitoring the excavation and support of deep foundations. To verify the accuracy of the simulation results, the Anhui Wuhu City Damao Interchange project was used as a case study. The project’s engineering overview was simulated concurrently, with wireless sensors strategically placed at various positions during the different excavation and support stages, as illustrated in Figure 11. The monitored parameters are listed in Table 4. The real-time data for the horizontal displacement of the excavation support structure, the surrounding ground settlement, and the bottom heave were collected, as shown in Figure 12. A comparison of the on-site monitoring data and the results of the simulation analyses based on the fluid–structure interaction was made, as depicted in Figure 13.

According to Figure 12, during the initial stage of dewatering, the horizontal displacement of the support piles changes minimally. At the beginning of the excavation, the top of the support piles starts to tilt toward the excavation, with little impact on the pile bottom. As the excavation depth increases, the top of the support piles tilts further towards the excavation, reaching a maximum value of 37.65 mm approximately 7.5 m from the top of the pile, which is below the warning threshold of 41.771 mm. The curve of ground settlement is relatively gentle during the initial dewatering of the excavation. With increasing excavation depth, the ground settlement further increases, reaching a maximum of −29.815 mm at a distance of 5 m from the excavation after 90 days, which is below the warning threshold of −32.682 mm. Overall, the heave at the symmetrical center of the excavation bottom continues to increase. The symmetrical center of the excavation bottom is initially sinking during the initial dewatering phase. As the excavation depth increases, the heave at the excavation bottom continues to expand, reaching a maximum value of 19.6 mm at point LZC2, which is below the warning threshold of 21.097 mm. All the measured values are below the warning threshold, indicating that the excavation is in a safe and stable state.

From Figure 13a, it is observed that the overall trend of the three curves is similar, with smaller values at the top and bottom and larger values in the middle. The displacement values when considering the fluid–structure interaction are the largest, followed by the measured displacement values, and the values that do not consider the fluid–structure interaction are the smallest. The measured curve closely matches the curve that considers the fluid–structure interaction, with the horizontal displacement maximum occurring at the middle position of the support pile. The maximum horizontal displacement when considering the fluid–structure interaction is 41.771 mm, the maximum measured displacement is 37.65 mm, and the maximum displacement without considering the fluid–structure interaction is 26.457 mm. The error between the maximum values when considering the fluid–structure interaction and the measured values is 4.121 mm, with an error rate of 10.9%, while the error between the maximum values without considering the fluid–structure interaction and the measured values is 11.193 mm, with an error rate of 29.7%.

In Figure 13b, the trends of the three curves are similar, starting out large and decreasing afterward. The settlement values when considering the fluid–structure interaction are the largest, followed by the measured settlement values, and the values that do not consider the fluid–structure interaction are the smallest. Similar to the horizontal displacement, the measured curve closely matches the curve that considers the fluid–structure interaction, with the settlement maximum occurring at a distance of about 5 m from the excavation. The maximum settlement when considering the fluid–structure interaction is −32.682 mm, the maximum measured settlement is −29.815 mm, and the maximum settlement without considering the fluid–structure interaction is −19.515 mm. The error between the maximum values when considering the fluid–structure interaction and the measured values is 2.867 mm, with an error rate of 9.6%, while the error between the maximum values without considering the fluid–structure interaction and the measured values is 10.3 mm, with an error rate of 34.5%.

From Figure 13c, it can be seen that the maximum heave values at the various points are not significantly different, with the maximum heave occurring at 19.6 mm and the minimum heave at 16.5 mm. All the measured values are below the heave values that consider coupled effects, with an error of 1.497 mm and an error rate of 7.6%. The error between the maximum values without considering the coupled effects and the measured maximum values is 6.292 mm, with an error rate of 32.1%.

Through the analysis of Figure 13, it can be concluded that the deformation values of the excavation are all below the construction monitoring warning values. The measured data closely align with the finite element simulation data, indicating that the sensors used in this experiment have minimal errors and high accuracy. In terms of the horizontal displacement, the maximum error between the simulated and measured values is 4.121 mm, with an error rate of 10.9%. For the ground settlement, the maximum error is 2.867 mm, with an error rate of 9.6%. Regarding the excavation bottom heave, the maximum error is 2.47 mm, with an error rate of 7.6%. These errors may originate from sensor zero offset errors, nonlinearity errors, temperature influence errors, mechanical vibration errors, and sampling rate errors. Further exploration is needed to delve into the reasons for these errors. In conclusion, a numerical simulation based on fluid–structure interaction theory exhibits high accuracy and practicality, providing new theoretical and data support for subsequent excavation projects.

## 6. Conclusions

This article explores the deformation patterns of an excavation support system, conducting simulations and analyses of the different support forms, water levels, permeability coefficients, excavation soil layers, and excavation sequences. This study obtained deformation results for excavation support under coupled effects and warning values for excavation deformation under coupled field effects, as well as the optimal dewatering scheme. The accuracy of the simulations was verified through the Damao Interchange Project in Wuhu, Anhui Province. The main conclusions are as follows:

(1) Different support forms significantly impact the horizontal displacement and surface settlement of the support structure, with minor effects on the bottom heave. The pile-supported system exhibits better stability than the external anchor-supported system, resulting in a 34.5% reduction in the overall deformation.
(2) In the pile-supported system, with a constant permeability coefficient, the horizontal displacement, surface settlement, and bottom heave are positively correlated with the water level, with the water level having the greatest impact on the surface settlement. In cases of a constant water level, an increase in the permeability coefficient leads to a larger horizontal displacement, surface settlement, and bottom heave. The permeability coefficient has the greatest impact on the bottom heave.
(3) In the pile-supported system, high water levels, high permeability coefficients, and multiple soil layers are the most unfavorable factors for excavation deformation. The order of the factors influencing excavation deformation is water level (35.5%) > permeability coefficient (17.62%) > excavation soil layer (11.4%). The optimal dewatering scheme for excavation construction is to lower the water level in the excavation to 0.5 m below the excavation surface.
(4) In the pile-supported system, sensor-monitored horizontal displacement, surface settlement, and bottom heave are all below the warning thresholds. The measured values closely align with the data from the simulations considering the fluid–structure interactions. Therefore, simulations based on fluid–structure coupling demonstrate higher accuracy and practicality. Hence, in actual excavation construction, the fluid–structure coupling mechanism should be thoroughly considered.

## Figures and Tables

**Table 1:** Physical and mechanical parameters of each soil layer.

	Physical Parameters	Fill	Silty Clay ①	Silty Clay Mixed with Silt	Silty Clay ②	Fully Weathered Muddy Sandstone	Strongly Weathered Muddy Sandstone
Name of Soil Layer							
Thickness (m)		5.5	3.8	5.8	10	1.1	3.8
γ (KN/m^3^)		17.5	19.5	18.5	19.6	19	21.3
$c^{\prime }$ (KPa)		5	51.7	24.6	51	35	45
$\varphi^{\prime }$ (°)		8	14.5	14.8	14.3	18	20
ψ (°)		0	0	0	0	0	0
$E_{50}^{ref}$ (MPa)		4.5	8.3	5.4	8.1	22	26
$E_{oed}^{ref}$ (KN/m^2^)		4.5	8.3	5.4	8.1	22	26
$E_{ur}^{ref}$ (KN/m^2^)		13.5	24.9	16.2	24.3	66	78

Note: (1). γ is the weight; $c^{\prime }$ is the effective cohesion; $\varphi^{\prime }$ is the effective internal friction angle; ψ is the shear angle; $E_{50}^{ref}$ is the main loading reference cutline modulus; $E_{oed}^{ref}$ is the main consolidator loading modulus; $E_{ur}^{ref}$ is the unloading–reloading reference modulus. (2). Silty Clay ①: grey yellow, containing kaolin, plasticity index 13.7. (3). Silty Clay ②: yellow, containing Fe, Mn nodules and kaolin, plasticity index 14.1.

**Table 2:** Support material parameters.

Name	E (GPa)	γ (KN/m^3^)
Piles	28	24
Internal support	210	78.5
Anchor rods	24	20

Note: E is the modulus of elasticity; γ is the heaviness.

**Table 3:** Table of enclosure unit parameters.

Name	E (GPa)	γ (KN/m^3^)
Floor to wall	28	24
Crown beam	30	25
Lattice columns	210	78.5
Steel support	210	78.5
Steel walings	210	78.5
Anchor rods	24	20

**Table 4:** Instrument monitoring items table.

Serial Number	Monitoring Instruments	Monitoring Projects	Measurement Point Frequency	Measurement Point Arrangement	Instrument Type	Measurement Accuracy
1	Oblique-gauge	Horizontal displacement	Twice a day during excavation	Center of the envelope	MAS-GCLI02 Tandem fixed inclinometer	±0.01°
2	Static level	Surface Settlement	Once every two days during excavation	Longitudinal average placement of foundation pits	TUH01-Ultrasonic Static Leveling Instrument	±0.1%FS
4	Gyroscope	Rising at the bottom of the pit	Twice a day during excavation	Pit overall arrangement of 12	ML7600-IMU	±0.01° ±0.01 g ±0.01°/s

## Data Availability

The data presented in this study are available on request from the corresponding author.
